# ACW-02 an Acridine Triazolidine Derivative Presents Antileishmanial Activity Mediated by DNA Interaction and Immunomodulation

**DOI:** 10.3390/ph16020204

**Published:** 2023-01-29

**Authors:** Sonaly Lima Albino, Willian Charles da Silva Moura, Malu Maria Lucas dos Reis, Gleyton Leonel Silva Sousa, Pablo Rayff da Silva, Mayara Gabriele Carvalho de Oliveira, Tatiana Karla dos Santos Borges, Lucas Fraga Friaça Albuquerque, Sinara Mônica Vitalino de Almeida, Maria do Carmo Alves de Lima, Selma Aparecida Souza Kuckelhaus, Igor José dos Santos Nascimento, Francisco Jaime Bezerra Mendonca Junior, Teresinha Gonçalves da Silva, Ricardo Olímpio de Moura

**Affiliations:** 1 Programa de Pós Graduação em Inovação Terapêutica, Universidade Federal de Pernambuco, Recife 50670-901, Brazil; 2 Laboratório de Desenvolvimento e Síntese de Fármacos, Departamento de Farmácia, Universidade Estadual da Paraíba, Campina Grande 58429-500, Brazil; 3 Programa de Pós Graduação em Ciências Farmacêuticas, Universidade Estadual da Paraíba, Campina Grande 58429-500, Brazil; 4 Programa de Pós Graduação em Química, Universidade Federal Rural do Rio de Janeiro, Seropédica 23890-000, Brazil; 5 Programa de Pós Graduação em Produtos Naturais, Sintéticos e Bioativos, Universidade Federal da Paraiba, João Pessoa 58051-900, Brazil; 6 Área de Morfologia, Faculdade de Medicina—UnB, Universidade de Brasília, Campus Darcy Ribeiro/Asa Norte, Brasília 70910-900, Brazil; 7 Laboratório de Imunologia Celular, Área de Patologia, Faculdade de Medicina, Campus Darcy Ribeiro, Brasília 70910-900, Brazil; 8 Faculdade de Ciências, Educação e Tecnologia de Garanhuns (FACETEG), Universidade de Pernambuco, Garanhuns 55290-000, Brazil; 9 Laboratório de Química e Inovação Terapêutica, Departamento de Antibióticos, Universidade Federal de Pernambuco, Recife 50670-901, Brazil; 10 Laboratório de Síntese e Vetorização de Moléculas, Universidade Estadual da Paraíba, João Pessoa 58071-160, Brazil

**Keywords:** leishmaniasis, immunomodulation, DNA binding, molecular docking, molecular dynamics

## Abstract

The present study proposed the synthesis of a novel acridine derivative not yet described in the literature, chemical characterization by NMR, MS, and IR, followed by investigations of its antileishmanial potential. In vitro assays were performed to assess its antileishmanial activity against *L. amazonensis* strains and cytotoxicity against macrophages through MTT assay and annexin V-FITC/PI, and the ability to perform an immunomodulatory action using CBA. To investigate possible molecular targets, its interaction with DNA in vitro and in silico targets were evaluated. As results, the compound showed good antileishmanial activity, with IC_50_ of 6.57 (amastigotes) and 94.97 (promastigotes) µg mL^−1^, associated with non-cytotoxicity to macrophages (CC_50_ > 256.00 µg mL^−1^). When assessed by flow cytometry, 99.8% of macrophages remained viable. The compound induced an antileishmanial effect in infected macrophages and altered TNF-α, IL-10 and IL-6 expression, suggesting a slight immunomodulatory activity. DNA assay showed an interaction with the minor grooves due to the hyperchromic effect of 47.53% and Kb 1.17 × 10^6^ M^−1^, and was sustained by docking studies. Molecular dynamics simulations and MM-PBSA calculations propose cysteine protease B as a possible target. Therefore, this study demonstrates that the new compound is a promising molecule and contributes as a model for future works.

## 1. Introduction

Leishmaniasis is a parasitosis caused by macrophage infection of protozoa from the genus Leishmania, transmitted to mammal host through the bite of blood-feeding females of phlebotomine sand flies [1]. It has been recognized as one of the main neglected tropical diseases (NTDs) of global health concern, estimating that over one billion people are living in endemic areas at risk of infection. Accordingly, each year more than two million new cases occur, associated with high levels of morbidity and mortality [2,3].

Leishmania parasites exist in two forms during their life cycle (amastigotes and promastigotes) and are capable of manifesting three main clinical forms, generally classified as cutaneous leishmaniasis (CL), mucocutaneous leishmaniasis (MCL) and visceral leishmaniasis (VL, also known as kala-azar). Depending on the parasite species and the cellular immune mechanisms of the host, different clinical, histopathological and immunopathological manifestations are developed [4,5].

The immune mechanism response of the host to the infection relies on myeloid cells such as dendritic cells and macrophages, which develops a complex relation [6]. Leishmania protozoa use macrophages as important host cells for establishing the infection. In order to adapt, the parasite regulates phagosome maturation, promoting the alteration of macrophage defenses such as oxidative damage, antigen presentation, immune activation and apoptosis, while there is an increase in nutrient availability, causing the macrophage to become a favorable environment for its growth and avoiding destruction [7]. 

Current chemotherapy includes drugs such as sodium stibogluconate, meglumine antimoniate, amphotericin B, paromomycin, and pentamidine. These, however, have limitations regarding the emergence of resistant strains, high costs, prolonged administration, and the manifestation of side-effects such as nephrotoxicity, ototoxicity, and hepatotoxicity [8,9]. In this sense, the development of new candidates for leishmanicidal drugs that are more effective against parasites, associated with less toxicity to the human host, stands out as a priority.

In this sense, the acridine nucleus has demonstrated to be an eligible scaffold due to its potential as a chemotherapeutic precursor [10]. As an antiparasitic acridine compound of clinical application, mepacrine stands out due to its wide spectrum of biological action, with its main application in the treatment of malaria in chloroquine-resistant strains. Therefore, several studies aimed at obtaining leishmanicidal compounds are based on the chemical structure of this compound, maintaining the 9-amino-2-methoxy-6-chloroacridine core while altering moieties linked to the 9-amino groups [11], as observed in the study developed by Serafim et al. [12], in which the authors have synthesized and evaluated the antipromastigote activity of thiophene-acridine derivatives against *L.* (L.) *amazonensis* strains, highlighting compounds ACS01 and ACS02 as potent and selective, with IC_50_ values of 9.60 and 10.95 µM against antimony-sensitive strains, and 14.83 and 16.36 µM against resistant strains, respectively. Furthermore, both molecules interacted with DNA with binding constants of 10^4^ M^−1^, in which the activity is attributed to the 6-chloro-2-methoxy-acridine moiety, as also previously reported by Zhang et al. [13].

Based on these previous data, our group proposed the synthesis of a novel acridine derivative containing the 6-chloro-2-methoxy-acridine moiety, through a rational strategy of molecular design based on a privileged structure, linked to a triazolidine-dithione nucleus obtained through an intramolecular spontaneous cyclization, followed by in silico and in vitro evaluation of its antileishmanial activity, mechanism of action through DNA interaction and immunomodulatory properties.

## 2. Results

### 2.1. Synthesis and Characterization

**Scheme 1:**
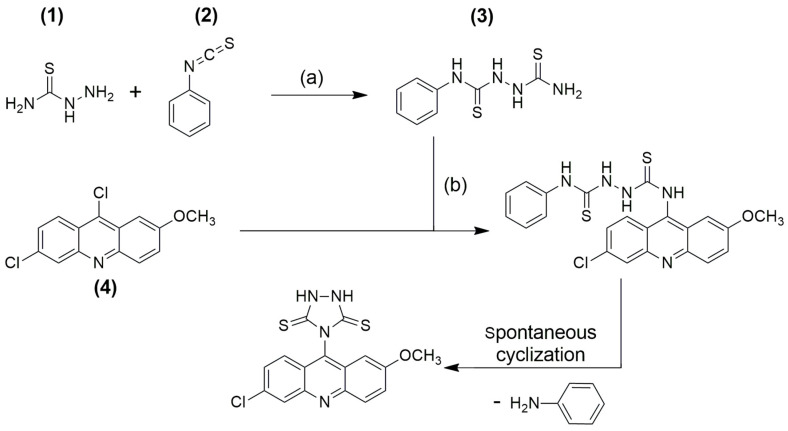
Synthesis of triazolidine acridines derivative. Reagents and conditions: (**1**) Thiosemicarbazide; (**2**) Isothiocyanate; (**3**) *N*-phenylhydrazine-carbothioamide; (**4**) 6,9-Dichloro-2-methoxyacridine; (a) EtOH, 50 °C; (b) EtOH, HCl, 78 °C.

In order to develop a new compound for the treatment of leishmania, a novel triazolidine acridine derivative was synthesized, obtained accidentally by spontaneous cyclization from a coupling reaction between two scaffolds: thiosemicarbazide and disubstituted 9-chloro-acridine. Scheme 1 outlines the synthesis with the sequence of the reactions to obtain compound ACW-02. The target compound was obtained in two steps, as described in the experimental section. The synthesized compound is being reported for the first time and its structure was confirmed by ^1^H NMR and ^13^C NMR spectroscopy, infrared spectroscopy and mass spectrometry, where the results were in agreement with the described structure. These data are present in the Supplementary Material (Figures S1–S4).

Through ^1^H NMR analysis, it was possible to identify a singlet for two symmetrical hydrogens with displacement at δ 12.79 ppm, correspondent to the triazolidine NH, corroborating with the findings of Tiwari et al. [14], which observed a peak in δ 12.48 ppm referring to triazolidine NH. Peaks characteristic of singlet (s), doublet (d), double doublet (dd), double triplet (dt) and multiplet (m) with displacements ranging between δ 7.36–8.83 ppm have been observed, indicative of the acridine ring. Furthermore, a singlet with an integral for three hydrogens in δ 3.88 ppm was recognized, correspondent to the OCH_3_ attached to the acridine ring. The ^13^C NMR spectrum also confirmed the structure by the presence of signals correspondent to the acridine nucleus, but mainly by the presence of thiocarbonyl groups, which showed symmetric displacement at δ 194.64 ppm, just as it was observed by Tiwari et al. [14]. Additionally, a peak in δ 55.86 ppm relative to OCH_3_ attached to the acridine ring was observed.

Infrared results also assisted in the structural characterization of the novel triazolidine acridine derivative. The following bands were observed: 3242–3147 cm^−1^, suggestive of axial deformation of secondary N-H; 3085–3056 cm^−1^, suggestive of aromatic C-H axial deformation; 1621 and 1482 cm^−1^, characteristic of C=C_Ar_ vibration; 1208 cm^−1^, suggesting the axial deformation vibration of C=S bound to nitrogen; 1197 cm^−1^, suggestive of asymmetric axial deformation of C=C-O attributed to the methoxyl group attached to the acridine ring. Ultimately, mass spectrometry (MS) was useful to confirm the structure of the novel synthesized compound, exhibiting the result of m/z = 375.81.

### 2.2. Antileishmanial and Cytotoxic Activity Evaluation

The antileishmanial potential of compound ACW-02 was explored in vitro against amastigotes and promastigotes forms of *Leishmania amazonensis*, responsible for causing the clinical cutaneous form of the pathology. For this purpose, Amphotericin B, a well-known commercial drug widely used in the treatment, was used as a positive control. It was observed that the synthesized compound showed good activity against the amastigote strain, with an IC_50AMA_ of 6.57 µg mL^−1^. Nonetheless, against the promastigote strain, a satisfactory result was not obtained (IC_50PRO_ = 94.97 µg mL^−1^). These results are presented in Table 1.

As for the cytotoxic effect, the synthesized compounds did not show cytotoxicity against JJ74 macrophage strains up to the highest concentration evaluated (256.00 μg mL^−1^). Thus, the compounds ACW-02 showed high rates of selectivity index for the amastigote forms of the parasite, with a value of >38.94. This contrasts with the results obtained for amphotericin, which, although it is a potent drug, presents a high level of toxicity, as already described in the literature [15] and can be observed in the low selectivity index (1.05) obtained.

Additionally, in the evaluation of the cytotoxic effect of the synthesized compound on macrophages, indications of immunomodulatory activity were observed through the identification of its stimulatory effects on the proliferation of such cells. The is quantified in EC_50_ value in Table 1 (EC_50_ = 9.46 μg mL^−1^). Therefore, in order to clarify the effects of the synthesized compound on macrophages and the possibility of an immunomodulatory effect, further studies were carried out.

### 2.3. Cytotoxicity Evaluation in Macrophages by Annexin V-FITC/PI Double Staining Assay

**Figure 1:**
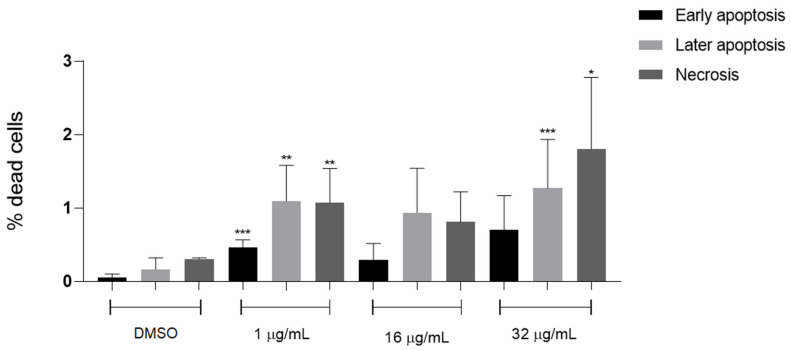
Effect of ACW-02 on J774 macrophages through the annexin V-FITC/PI method after 2 h. Asterisks represent statistically differences between treated and untreated macrophages; * *p* < 0.05; ** *p* < 0.01; *** *p* < 0.001. ANOVA followed by Tukey’s multiple comparisons test.

In order to understand in more detail the effect of the synthesized compound on the J774 macrophages, a cell cycle evaluation assay using the annexin V-FITC/PI method was performed. The results reaffirm the compound’s non-cytotoxicity to macrophages, with the percentage of living cells corresponding to approximately 98%, up to the highest concentration tested. The results are shown in the graph in Figure 1.

As demonstrated, in the absence of the compound, all cells were viable as the percentage of initial, late apoptosis and necrosis was 0%. In contrast, in a concentration of 1 µg mL^−1^, the percentage of cells in initial, late apoptosis and necrosis was 0.46, 1.09 and 1.07, respectively. When increased to the concentration of 16 µg mL^−1^, there is a decrease in the percentages, these being 0.29, 0.94 and 0.81, respectively. Finally, at a concentration of 32 µg mL^−1^, the percentages increase to 0.70, 1.27 and 1.8, respectively. In comparison to negative control, which has shown values of 0.06, 0.17 and 0.31%, ACW-02 exhibits approximate values, highlighting its viability as non-cytotoxic to macrophages.

### 2.4. Determination of the Microbicidal Effect in Cultures of J774 Macrophages Infected with L. amazonensis

Previous studies provided information about the antileishmanial activity of the evaluated compound associated with a possible immunomodulatory activity on macrophages. Given the importance of this cell for the progression of the pathogenesis in question, this possibility represents a pertinent association of mechanisms. Therefore, the effect of ACW-02 on macrophages infected with *L. amazonensis* was evaluated, in order to verify its immunomodulatory potential. The results are shown in Table 2.

Our results evaluated by the J774 macrophage infection index showed the microbicidal potential of ACW-02. In comparison to infected and untreated cells (0 µg mL^−1^), it was observed that the addition of ACW-02 reduced the infection rate in all concentrations (1, 16 or 32 µg mL^−1^), due to the decrease in the percentage of infected macrophages and the number of Leishmania ingested by macrophages (Table 2).

**Figure 2:**
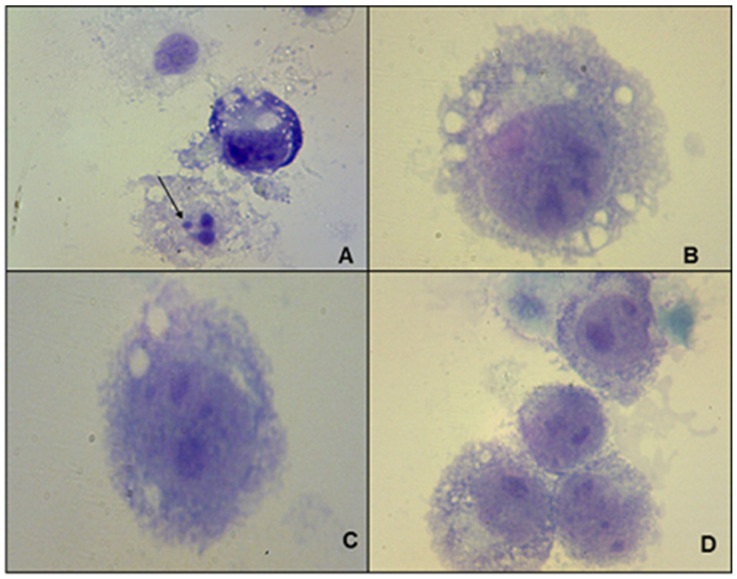
Macrophages J774 experimentally infected by *Leishmania amazonensis.* (**A**) negative control (0 µg mL^−1^), (**B**) ACW-02 treated in concentration of 1 µg mL^−1^, (**C**) 16 µg mL^−1^ and (**D**) 32 µg mL^−1^. The black arrow indicates the presence of the macrophage-internalized *L. amazonensis* amastigote. Stain: Giemsa 10%. Bars = 10 µm.

The microbicidal effect of ACW-02 is shown in Figure 2. The low infection rate, especially at the lowest concentrations of ACW-02 (1 or 16 µg mL^−1^), possibly interfered with the functionality of macrophages, thus favoring the elimination of parasites.

### 2.5. Assessment of Cytokine Production by Macrophages

**Figure 3:**
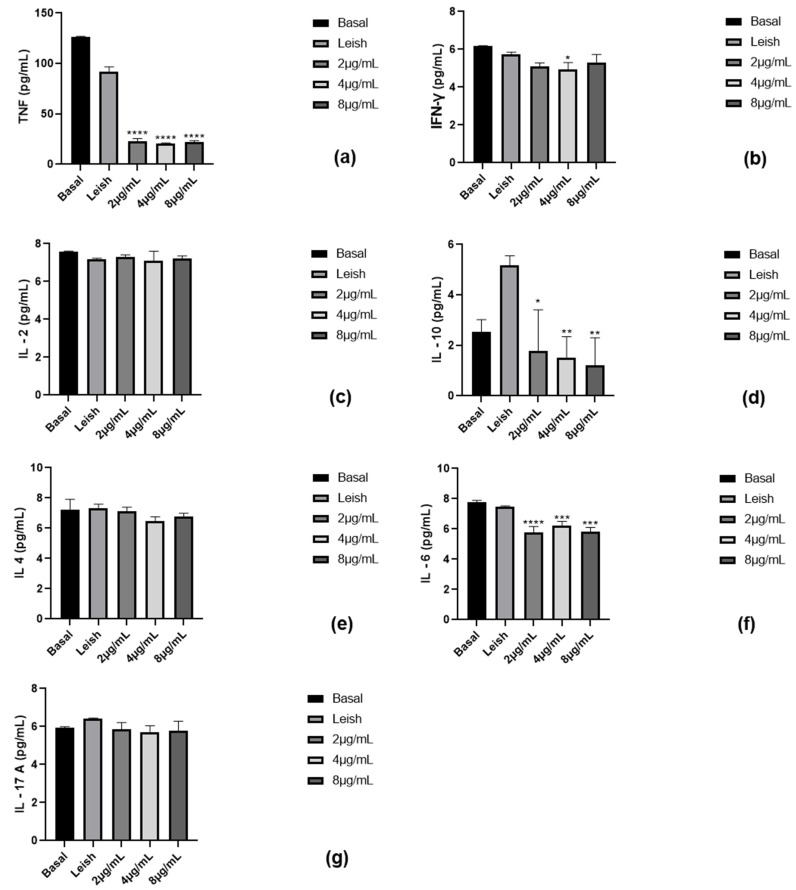
Assessment of Th1 immune response ((**a**) TNF-α, (**b**) IFN-γ, (**c**) IL-2), Th2 ((**d**) IL-10, (**e**) IL-4, (**f**) IL-6) and Th17 ((**g**) IL-17A). Basal: Macrophages uninfected and untreated. Leish: Macrophages infected with *L. amazonensis* and untreated. Asterisks represent statistically differences between treated and infected macrophages untreated; * *p* < 0.05; ** *p* < 0.01; *** *p* < 0.001; **** *p* < 0.0001. ANOVA followed by Tukey’s multiple comparisons test.

Evaluations were made concerning the effect of the compound ACW-02, at concentrations 2, 4, and 8 µg mL^−1^, on the modulation of the Th1 (T helper 1), Th2 (T helper 2), and Th17 (T helper 17) response through the expression of the respective cytokines: TNF-α (Tumor Necrosis Factor-α), IFN-γ (Interferon-γ), IL-2 (Interleukin-2), IL-10 (Interleukin-10), IL-4 (Interleukin-4), IL -6 (Interleukin-6) and IL-17A (Interleukin-17A). The response expressed by the basal macrophage (untreated and uninfected) and macrophage infected with *L. amazonensis* (untreated) were used as negative and positive controls, respectively (Figure 3).

The progression of the infection caused by Leishmania protozoa is directly related to the immune response developed by the host, especially the T-cell mediated immunity and the expression of pro-inflammatory and/or anti-inflammatory cytokines, that can result in an asymptomatic, self-healing, or chronic leishmaniasis. The cellular immunity generated by the Th1 is considered to be the key mediator of resistance to Leishmania, as they are associated with the secretion of pro-inflammatory cytokines (e.g. TNF-α, IFN-γ, IL-2) that activate the MΦ killing machinery, leading to parasite inactivation [16,17,18]. Nevertheless, according to the results obtained in our experiments, there is a significant decrease in the expression of TNF-α (Figure 3a) with the addition of the acridine compound ACW-02, at the lowest up until the highest concentration evaluated, thus favoring an anti-inflammatory action. Further, the effect of the compound on IFN-γ and IL-2 cytokines was not significant.

On the other hand, cytokines expressed by Th2 cells (e.g. IL-4, IL-6, IL-10) are related to an anti-inflammatory action, as they are capable of suppressing the host’s immune system, causing macrophage depletion and, consequently, promoting development of the intracellular forms of the parasite and disease progression [17]. According to our findings, a decrease in IL-10 expression is observed (Figure 3d), approaching basal values at the lowest concentration tested (2 µg mL^−1^). Moreover, there is a significant decrease in IL-6 expression at all concentrations evaluated. Opposed to the previous results, the decrease in the expression of an anti-inflammatory interleukin will favor a pro-inflammatory effect, that is, a leishmanicidal action. Results found for the other evaluated cytokine, IL-4, were not significant.

Lastly, Th17 is involved in the production of IL-17, a highly pro-inflammatory cytokine with significant effects on stromal cells, resulting in the recruitment of leukocytes and forming an interaction between innate and adaptive immunity [16,19]. However, according to the results obtained, the compound ACW-02 does not show significant activity on the cytokine IL-17A (Figure 3g).

### 2.6. Reactive Oxygen and Nitrogen Species Evaluation

The production of reactive oxygen (ROS) and nitrogen (RNS) species is of significant relevance to disease progression. The activation of macrophages as a response to an infectious process induces the overproduction of reactive oxygen species (ROS), including superoxide, hydrogen peroxide, hydroxyl radicals, and reactive nitrogen species (RNS), such as nitric oxide (NO). ROS and RNS exhibit high microbicidal capacity, as these species can generate oxidative damage to parasite’s biomolecules, such as lipids, proteins, and DNA, leading to loss of membrane integrity, defective replication, and ultimately cell death [20,21].

**Figure 4:**
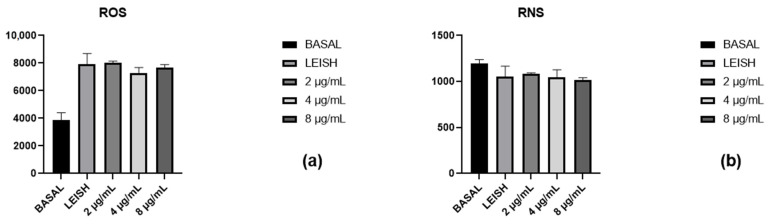
Evaluation of the effect of ACW-02 on the production of reactive (**a**) oxygen and (**b**) nitrogen species. Basal: Macrophages uninfected and untreated. Leish: Macrophages infected with *L. amazonensis* and untreated. ANOVA followed by Tukey’s multiple comparisons test.

In order to evaluate the ability of the synthesized compound to induce the formation of reactive species, evaluations were carried out that showed that the compound under evaluation at concentrations 2, 4, and 8 µg mL^−1^ did not significantly affect the production of ROS, when compared to the infected and untreated macrophages, as shown in Figure 4a.

Similarly, at the same concentrations (2, 4, and 8 µg mL^−1^), ACW-02 presented no significant alteration on RNS production in comparison to infected and untreated macrophages, as shown in Figure 4b.

### 2.7. Interaction with DNA

In order to propose a potential target that justifies the antileishmanial action of the acridine compound ACW-02, interaction studies with the DNA molecule were performed.

The UV–vis absorption spectra of ACW-02 showed significant absorption in the region between 400–500 nm. This derivative exhibited, in the absence of ctDNA, maximum peak at 460 nm, while in the presence of ctDNA, it presented a maximum peak of 461 nm. The maximum absorption wavelengths of the compound in the absence and presence of ctDNA can be observed in Table 3.

**Figure 5:**
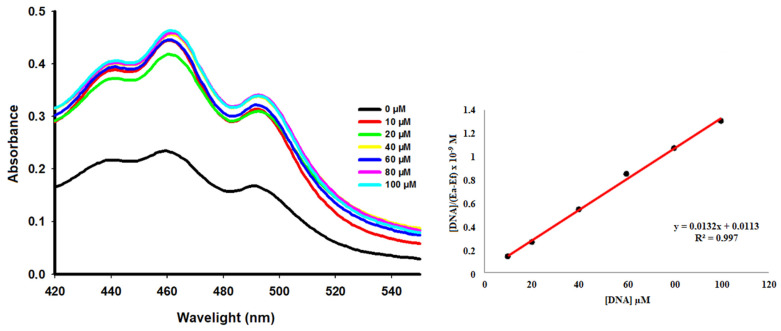
Absorption spectrum (10 μM) in the absence and presence of increasing amount of ACW-02.

In these studies, the model of McGhee and Von Hippel [22] was used to estimate the binding constant (Kb) from the spectrophotometric data. The interaction by intercalation can be characterized by the hypochromic effect and by the wavelength alteration for the red region (bathochromic effect) [23,24,25,26], whereas the occurrence of hyperchromism with little or no redshift is associated with electrostatic attachment or interaction with DNA groove (major or minor) [27,28]. According to the absorption results, ACW-02 presented hyperchromism of 47.23% and Kb (M^−1^) = 1.17 × 10^6^ in the presence of ctDNA (Figure 5).

According to the data, ACW-02 presented characteristics suggestive of interaction either by intercalation or by interaction with DNA grooves due to its Kb value. Kb values for intercalation complexes with DNA ranges from 1 × 10^4^ to 1 × 10^6^ M^−1^, while Kb values of groove binders range from 1 × 10^5^ to 1 × 10^9^ M^−1^ [29]. To confirm this effect, molecular docking studies were performed.

#### Docking Studies of Interaction with DNA

**Figure 6:**
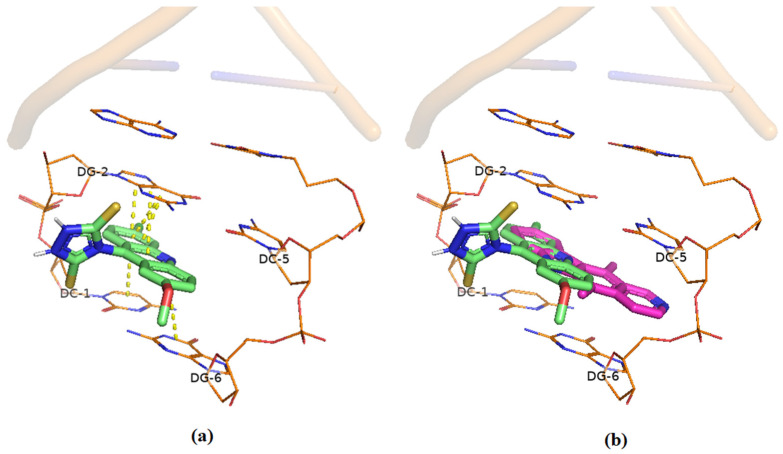
Proposed fit for ACW-02 in DNA (PDB ID: 1Z3F). (**a**) on the left, the proposed molecular fit of ACW-02 in DNA; hydrophobic interactions with DNA bases are shown in yellow dashed lines. (**b**) overlap of ellipticin fitting (magenta) with the ACW-02 (green).

The DNA intercalation capacity of ACW-02 in silico was evaluated using the crystallographic fitting model available in the PDB for Ellipticin (PDB ID: 1Z3F). Figure 6 shows the highest-scoring ACW-02 conformer (71.77) after the molecular fit study. The acridine portion of the compound indicated more affinity to intercalate between the nitrogenous bases of DNA through π-π electron stacking. This stacking limit between the bases was delimited by the steric hindrance of the cyclic triazolidine-derived portion in the structure. As proposed in spectroscopic studies, molecular anchoring also indicated the ability of double interaction with DNA, with the triazolidine fragment capable of interacting with the side chains of the macromolecule. Although ACW-02 had a lower score than ellipticin (75.68), they showed great similarity in terms of pose and intermolecular interactions performed with DNA (Figure 6), highlighting the ability of the acridine derivative for new interactions in the receptor.

Although these results indicate the ability of the compound to interact with DNA, this is probably not the only mechanism of action performed by the compound under evaluation, since there is no structural difference between the DNA molecules of the parasite and the host that provides selectivity to the compound, and, consequently, justifies the entirety of the leishmanicidal action. For this reason, the possibility of new targets was evaluated in silico to investigate possible complementary mechanisms of action.

### 2.8. Theoretical Study of Possible Mechanisms of Action

#### 2.8.1. Molecular Docking

The high selectivity presented in the in vitro tests of ACW-02 against *Leishmania amazonensis* supported the in silico study of possible mechanisms of action involved in the survival of the parasite. Some metabolic pathways explored in the disease, such as: redox balance (Trypanothione reductase, TryR); regulation of DNA supercoiling by topoisomerases I (Topo I); and the enzymes involved in ergosterol biosynthesis (14-alpha-ester demethylase, CYP51) were explored by molecular docking [12,30,31,32]. Moreover, cysteine proteases, such as cysteine protease B from *Leishmania amazonensis* (CPBLa) are promising drug targets against parasitic diseases, essential for the biochemical processes and survival of parasites [33]. The crystallographic models used for the theoretical studies were obtained from the PDB and the CPBLa was built using homology modeling. Validations by redocking, when possible, were performed and presented acceptable results for the study [34]. The ACW-02 presented scores lower than the standards complexed to the targets studied in the docking (Table 4). However, information about the main residues and polar interactions intrinsic to the formation of the ligand-receptor complex were reproduced and indicated good affinities for the docking of ACW-02 in the studied targets; characteristics such as planarity and points of polar interactions in the cyclic triazolidine-derived portion were essential for the performance of the scaffold in this study. For example, in TryR (PDB ID: 4APN), polar interactions with residues such as Lys^61^ and hydrophobic interactions with residues Val^58^ and Leu^62^ are reported to be essential for docking in the enzyme. In this study, ACW-02 demonstrated the ability to reproduce these interactions and, additionally, pointed out other interactions with nearby residues in the aforementioned cavity [10,35]. Finally, interaction analysis CPBLa reveled H-bond with the key residues Cys^153^ and van der Waals with His^289^ and Gly^151^, proposing that the acridine derivative can perform on the cysteine protease as a drug target. 

**Figure 7:**
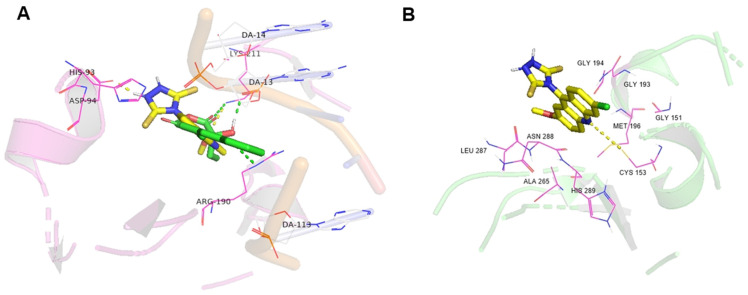
Docking analysis of Camptothecin (green) and ACW-02 (yellow) in LdTopo IB (PDB ID: 2B9S) (**A**), and ACW-02 with CPBLa (**B**). The polar interactions are highlighted as dashed lines in the respective colors of the derivatives studied.

Planar condensed fragments are commonly associated with mechanisms that directly or indirectly involve DNA, due to their validated intercalation capability, as visualized in aforementioned in silico and in vitro studies with DNA performed in this paper [36,37]. The docking results found suggest that Topo IB (PDB ID: 2B9S) is a possible mechanism for ACW-02. Due to the absence of a co-crystallized ligand to the topoisomerase I target, Camptothecin (CPT) was used as a standard compound, given its elucidated mechanism of action as a topoisomerase I inhibitor [10]. The intrinsic polar interactions of Camptothecin in docking with LDTopoI (63.98) were reproduced with residues Arg^190^ and Lys^211^ [38]. As in the docking with DNA, the cyclic portion linked to acridine induced a steric restriction for an improved stacking between DNA topoisomerase I bases (Figure 7A), however, this same fragment was responsible for additional interactions with the enzymatic portion of topo I, which justifies the similarity between the scores found for ACW-02 and Camptothecin.

On the other hand, the docking analysis of the ACW-02 in CPBLa (Figure 7B) has several interactions that can propose this drug target. First, acridine nitrogen interacts with the Cys^153^ by H-bond, and the aromatic ring is positioned to perform van der Waals interactions with the catalytic residue of His^289^. In addition, interactions amide-π Stacked are formed with the residue Asn^288^ and π-alkyl with Leu^287^ and Ala^288^. Finally, repulsive interactions are observed with hydrophobic residues such as Gly^151^, Gly^193^, and Gly^194^. Similar interactions were found in docking analyses of the standard compound. Finally, the most effective fit score of ACW-02 compared with the standard (50.97 and 47.77, respectively) and most effective interactions propose the CPBLa as the possible drug target. Thus, to confirm our findings, MD simulations and MM-PBSA calculations were performed and discussed in the following topics. Two-dimensional figures of these dockings are present in the Supplementary Material (Figures S7–S10).

**Table 4:** Molecular docking results of ACW-02 against potential antileishmanial targets.

	4APN (TryR)	3L4D (CYP51)	2B9S (Topo I)	CPBLa
**ACW-02**	54.22	58.74	60.34	50.97
Standard *	67.58	78.77	63.98	47.77
**RMSD**	0.76	1.02	-	-

* Ligand co-crystalized in 4APN for TryR; ligand co-crystalized in 3L4D for CYP51; Camptothecin for Topo I; and compound **1c** [39] for CPBLa.

#### 2.8.2. Molecular Dynamics (MD) Simulations

**Figure 8:**
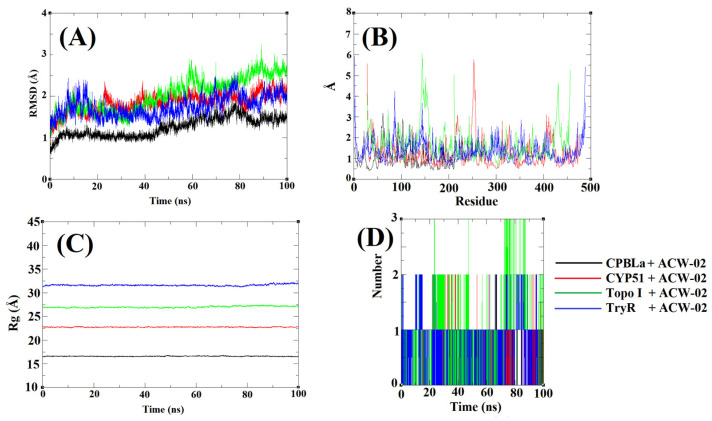
MD simulations plots of (**A**) RMSD; (**B**) RMSF, (**C**) Rg; and (**D**) h-bonds of the CPBLa; CYP51; Topo I and TryR in complex with ACW-02.

Molecular dynamics simulations were performed in trajectory of 100 ns (Figure 8) to confirm the drug target of ACW-02. The RMSD plot (Figure 8A) reveals the best stability of ACW-02 in complex with CPBLa, showing values lower 2 Å. On the other hand, the compound in complex with the other targets shows up to 3 Å. In addition, the RMSF plots (Figure 8B) reveal lower fluctuations of the residues for the complex with CPBLa, suggesting the best stability of the complex. The stiffness and protein compaction is shown in the R_g_ plot (Figure 8C), in which better protein compaction for the complex with CPBLa (between 15 and 16 Å), different of the other complexes that presented values higher than 15. Finally, the compounds showed similar h-bonds interactions around the trajectory (Figure 8D), up to 2 for the complexes with CPBLa, CYP51, TryR, and up to 3 for Topo I. The MD results are according to other reports and generate useful information that can be used to propose the complex stability and interaction with the targets [40,41,42]. Thus, these findings suggest the best stability of the ACW-02 with the CPBLa and can be a possible drug target.

#### 2.8.3. MM-PBSA Calculations

MM-PBSA calculations using the MD simulations trajectories were used to re-score the affinities energies and determine the attractive forces involved in the formation of the ACW-02 complexes against the proposed targets (Table 5). As observed in molecular docking studies, the affinity energy of ACW-02 against CPBLa by MM-PBSA (ΔG_bind_ = −103.13 KJ/mol) was superior to the complex with other proposed targets. Furthermore, it is clear that van der Waals interactions are essential for complex formation (DGvdw = −151.75 KJ/mol) when compared to electrostatic forces (0.68 KJ/mol). The polar solvation and SASA energy show low solvation (61.09 and −13.15 KJ/mol, respectively), indicating more excellent permanence of the ligand in a hydrophobic environment free of water molecules when compared to its interaction with other targets. Finally, the results suggest CPBLa as one of the main targets of ACW-02.

## 3. Discussion

The compound under study is an unprecedented triazolidine-acridine derivative. The initial design of the compound contemplated the coupling reaction between the di-substituted acridine nucleus and the intermediate *N*-phenylhydrazine-carbothioamide, a thiosemicarbazide derivative, selected during the design stage due to the inherent potential of the fragment that gives it a broad spectrum of biological activity [43,44], including anti-protozoal action [45,46,47]. However, during the synthesis stage, the triazolidine-dithione nucleus was obtained through a nucleophilic substitution reaction between the carbothioamide intermediate and the acridine nucleus, followed by an intramolecular attack favored by the energy provided by the heating of the reaction and by the free protons in the reaction medium, inducing an autocyclization and a fragmentation of the aniline portion from the carbothioamide subunit, forming a new triazolidine-thione nucleus coupled to the substituted acridine. Thus, due to the novelty of the compound, it was decided to continue the studies by observing the contributions of this new ring to the acridine scaffold.

The reaction to obtain the final product occurred in an ethanolic and acidic medium, at reflux temperature, and was finalized by cooling the reaction to room temperature, followed by the addition of distilled water and precipitation of the product, subsequently filtrated. Ultimately, ACW-02 was elucidated by ^1^H and ^13^C NMR, infrared, and mass spectrometry techniques, showing signs that confirmed the proposed structure.

Therefore, given the potential of acridine compounds as chemotherapeutic agents, with several studies exploring and reporting their anticancer anticancer [13,48,49,50,51], antibacterial [52,53,54] and antiparasitic [12,55,56] properties, this study aimed to evaluate the potential antileishmanial activity of the new synthetically obtained compound, given the urgent need for efficient drugs for this purpose.

Leishmaniasis comprises the pathology caused by protozoa of the genus *Leishmania*. In its digenetic life cycle, the parasite assumes metacyclic promastigote and amastigote forms in the vertebrate host. The promastigote forms are initially inoculated into the host through the bite of the sandfly mosquito and, in the bloodstream, the parasites penetrate phagocytic cells through interactions between their surface molecules and macrophage receptors. In the phagocytic vacuole, these differentiate into the amastigote form and multiply intensely until the infected macrophage ruptures. These free amastigotes, in turn, infect other macrophages and continue the cycle [4,57]. Therefore, depending on the drug’s affinity for each parasitic form, different stages of the infection progression will be achieved. According to the studies carried out, the compound ACW-02 presented an IC_50_ value of 6.57 µg mL^−1^ against amastigote forms of *L. amazonensis*, and 94.97 µg mL^−1^ against promastigote forms of the same species.

Depending on the infecting species, different clinical manifestations may develop. The species under study, *Leishmania amazonensis*, is an important etiological agent that causes American cutaneous leishmaniasis (ACL) and is capable of establishing a broad spectrum of clinical manifestations, varying from: cutaneous, localized or diffuse, which are the most common; mucocutaneous; and even visceral leishmaniasis [58]. Thus, it is observed that the amastigote forms are more sensitive to the pharmacological action of ACW-02 than the promastigote forms, indicating the possible potentiality of the compound against these forms of *L. amazonensis* in the treatment of ACL.

Moreover, based on the report by Serafim et al. [12], the hybridization of the thiophene portion with the 6,9-dichloro-2-methoxy-acridine ring was beneficial to the evaluated antipromastigote activity against *L. amazonensis*, obtaining IC_50_ values between 9.62 and 69.11 µM. However, the hybridization of the non-substituted acridine ring to the thiophene portion caused a loss in activity, highlighting then the relevance of the substitution in the acridine ring by groups with positive mesomeric effect (–OCH_3_ and –Cl) for antileishmanial activity. As this study involves obtaining an unprecedented molecule that possesses the substituted acridine nucleus, the same as that used by Serafim et al. [12], it is supposed that this portion of the molecular structure of ACW-02 is associated with the antileishmanial activity presented.

Considering the importance of macrophages during the installation and progression of the *Leishmania* infectious process [59], studies involving the effects of ACW-02 on J774 macrophages were carried out. The determination of CC_50_ demonstrates that the compound did not show cytotoxicity up to the highest concentration tested (256.00 µg mL^−1^), and this result is confirmed in more detail through the evaluation of cytotoxicity in macrophages by Annexin V-FITC/PI double staining assay, which showed low percentages of cells (<2%) in initial, late apoptosis and necrosis after treatment with ACW-02 at concentrations of 1, 16, and 32 µg mL^−1^. Thus, evaluating the first result mentioned, the calculated selectivity index of ACW-02 to *L. amazonensis* amastigote cells was above 38.94, providing a safety margin.

Additionally, a stimulatory effect on macrophages was observed at a concentration of 9.46 µg mL^−1^ in preliminary studies and the possibility of the compound’s ability to play an immunomodulatory effect on macrophages was evaluated in more comprehensive studies. The determination of the microbicidal effect contributed to the supposed possibility of altering the functionality of the macrophage in the face of parasitic infection with the addition of the ACW-02 compound, due to the alteration in the parameters that evaluate the percentage of infected cells, the average of *Leishmanias* and percentage of infection, especially in lower concentrations (1 and 16 µg mL^−1^) and, accordingly, closer to the EC_50_ value obtained. Moreover, it is supposed that the highest concentration (32 µg mL^−1^) may have caused a supersaturation on the culture medium during the test, thus causing a modification in the solubility of the compound and consequent decrease in biological activity, due to the lower interaction of the compound with the infected macrophages.

To determine the influence of the compound directly on the components of the immune system, targeted studies were carried out. The control of the infection and disease progression caused by *Leishmania* sp. is associated with the generation of pro-inflammatory and anti-inflammatory immune responses. Stimulation of a Th1 response and the downmodulation of a Th2 response promotes macrophage activation and, consequently, assists in the host control of *Leishmania* parasite burden and clinical cure [18,60]. The results obtained in response to Th2 demonstrated a significant decrease in the expression of IL-10 and IL-6 cytokines, thus contributing to an antileishmanial effect. These results corroborate with the aforementioned studies and may justify the macrophage’s ability to perform a microbicidal effect against *Leishmania*. However, these results are not sufficiently conclusive, since there is also a significant decrease in TNF-α expression, which, on the other hand, exerts a pro-leishmania effect. Thus, the effect accomplished by reducing TNF-α is antagonistic to the one obtained by decreasing IL-10 and IL-6. 

Similarly, macrophage activation promotes the production of reactive oxygen and nitrogen species to induce oxidative damage to the parasite [20]. However, the results did not demonstrate an alteration in these components that would justify the performance of an immunomodulatory activity by ACW-02 at the concentrations evaluated (2–8 µg mL^−1^). Thus, due to the lack of results that establish the immunomodulatory effect as justification for the entirety of the antileishmanial activity, other pharmacological targets were evaluated in silico and in vitro.

The acridine nucleus, present in the structure of the compound ACW-02, stands out as a potential scaffold. Its biological activity can be attributed to the planarity of its aromatic structures, which can interact by intercalation or interactions with the DNA grooves alone, interfering in cellular properties [61,62,63]. In view of this potential mechanism of action of acridine compounds, the interaction of the compound ACW-02 with DNA was evaluated. 

The presence of aromatic groups without substituents may favor the interaction of the complex through strong hydrophobic or non-covalent interactions between the electron states of the chromophore and DNA base pairs, leading to a decrease in the transition energy π → π*. However, despite the acridine ring possible interactions, the hyperchromic effect observed in this compound may be due to the additional electrostatic interactions of the methoxyl (–OCH_3_), of the acridine ring, which has σ+ and π- effects, and of the chlorine atom (–Cl), which has σ+ and π+ effects, resulting in a reduction of the stacking of bases of this biomolecule [64,65,66].

In general, the non-substituted acridine nucleus presents intercalating activity [67,68] through π-π interactions with DNA base pairs. The insertion of the triazolidine nucleus, presenting electron acceptor groups (C=S) and electron donors/acceptors (–NH), may favor the stabilization of the supramolecular DNA-ligand complex, through electrostatic or non-covalent interactions [69,70,71]. Thus, favoring a possible intercalation and interaction with DNA grooves.

In silico molecular docking studies were used as a strategy to reaffirm the interaction with DNA through studies on the ellipticine-complexed DNA target (PDB ID: 1Z3F), demonstrating an ACW-02 score proximate to that of the ellipticine standard (71.77 and 75.68, respectively).

Aiming to elucidate possible molecular mechanisms that could contribute to the entirety of the antileishmanial action of the compound ACW-02 and justify its selectivity for the parasite, theoretical studies of molecular docking were performed in pharmacological targets exclusive and essential to the *Leishmania* (TryR, Topo I, CPBLa, and CYP51). The results demonstrated the in silico affinity of the compound to the targets and interactions with essential amino acid residues capable of propagating a tissue response. In view of these findings, important interactions of the compound ACW-02 are observed, especially against the topoisomerase I target, in which the compound presented a score value comparable to the control compound Camptothecin (60.34 and 63.98, respectively). This enzyme is responsible for the relaxation of supercoiled DNA that occurs during DNA replication and transcription processes, and its blockage can lead to cell death [72]. Therefore, topoisomerase I is a promising target for antileishmanial compounds since the enzyme is overpressed during Leishmania’s rapid life cycle and is structurally distinct from the human topoisomerase I [37].

On the other hand, ACW-02 reveled the best interaction against the CPBLa by molecular docking (fit score of 50.97 and 47.77 for the standard) and interactions with key residues (Cys^153^, His^289^, and Gly^151^). Similar to other cysteine protease inhibitors [73], the interactions with key residues are related to inhibiting the enzyme’s catalytic functions and preventing the parasite’s life cycle [74,75]. In addition, the best stability in MD simulations and great Δ*G*_bind_ through MM-PBSA calculations (−103.13 KJ/mol) propose the CPBLa as a possible drug target. Others works highlight these methods as essential to predict drug targets of leishmaniasis [76,77]. Furthermore, these findings are in agreement with other works that underline the acridine derivatives as promising cysteine protease inhibitors [78]. 

Moreover, these findings corroborate with in silico and in vitro results of interaction with DNA, suggesting the possibility of the compound performing a dual pharmacological activity, that is, being able to intercalate with DNA bases and inhibit the activity of the *Leishmania* topoisomerase I enzyme and CPBLa.

Therefore, further studies that support in the mechanistic elucidation that can justify the leishmanicidal effect of the compound, whether in silico, in vitro, or in vivo, are envisioned by our research group, especially aiming to clarify its activity against topoisomerase I and CPBLa from *Leishmania*, as well as the expansion of the chemical series to obtain new derivatives that may present more favorable results.

## 4. Materials and Methods

### 4.1. General Procedure for the Synthesis of Triazolidine Acridine Derivative

The intermediate *N*-phenylhydrazine-carbothioamide (**3**) was obtained through a nucleophilic addition reaction between thiosemicarbazide (**2**) and isothiocyanate (**1**), in ethanol at 50 °C. Then, 6,9-dichloro-2-methoxy acridine (Sigma Aldrich, Saint Louis, MO, USA) (**4**) was coupled to the intermediate *N*-phenylhydrazine-carbothioamide by an aromatic nucleophilic substitution reaction at 78 °C in ethanolic medium and acid, followed by spontaneous cyclization according to Scheme 1. The reaction was monitored by Analytical Thin Chromatography (CCDA), determining the end of the reaction. The reaction was filtered and the obtained crystals were washed with ice-cold distilled water, and then recrystallized in ethanol. The product was then analyzed by ^1^H and ^13^C NMR (Agilent NMR spectrometer, mod. Mercury Plus 500 MHz, OXFORD 300 magneto-NMR, Santa Clara, CA, USA), infrared spectroscopy (IRPrestige-21 Spectrophotometer, Shimadzu, Kyoto, Japan) and mass spectrometry (Shimadzu^®^ AXIMA series MALDI-TOF/MS, Kyoto, Japan).

#### 4-(6-Chloro-2-methoxyacridin-9-yl)-1,2,4-triazolidine-3,5-dithione (ACW-02)

Orange crystals. Formula: C_16_H_11_ClN_4_OS_2_; M.W.: 374.8610 g mol^−1^; Yield: 50%; Melting point: 282–284 °C; Rƒ: 0.50 (*n*-hexane/EtOAc 7:3). ^1^H NMR (500 MHz, DMSO-*d*6): δ 3.88 (3H, *s*, OCH3); 7.36 (1H, *dd*, J = 2.05 Hz; J = 9.05 Hz, H-12); 7.52 (1H, *dd*, J = 2.9 Hz; J = 9.05 Hz, H-01); 7.62 (1H, *d*, J = 9.1 Hz, H-06); 7.65 (1H, *d*, J = 2 Hz, H-14); 8.25 (1H, *d*, J = 2.9 Hz, H-03); 8.83 (1H, *d*, J = 2.9 Hz, H-11); 12.79 (2H, *s*, NH). ^13^C NMR (100 MHz, DMSO-*d*6): δ 194.64 (C-19, C-16); 156.34 (C-13); 138.50 (C-4); 136.27 (C-8); 132.48 (C-3); 131.33 (C-2); 130.46 (C-5); 127.57 (C-9); 126.32 (C-6); 126.96 (C-12); 120.90 (C-1); 117.82 (C-11); 108.43 (C-14); 55.86 (OCH_3_). IR (KBr, cm^−1^): 3242–3147 (N-H); 3085–3056 (C-H_Ar_); 1621–1482 (C=C_Ar_); 1482 (N-C=S); 1208 (C=S); 1197 (C=C-O). MALDI-TOF MS m/z [M+H]^+^: calculated = 374.0063; found = 375.0096.

### 4.2. Antileishmanial and Cytotoxic Activity

#### 4.2.1. Cultures of *L. amazonensis*

The amastigote cultures of *L. amazonesis* MHOM/BR/pH8 strain were kept under cryopreservation until transferred to NNN medium (Novy–MacNeal–Nicolle) and cultured at 26 °C until the parasites reached the log growth phase. Then, the suspension was transferred to Schneider culture medium (Sigma-Aldrich, St. Louis, MO, USA), supplemented with 10% inactivated fetal bovine serum and 0.2% gentamicin sulfate, at 26 °C, so that the parasites returned to the log phase of growth. After that stage, the promastigote forms were incubated at 37 °C, producing a growth curve and the formation of the axenic amastigote strains [79].

The promastigote forms of *L. amazonensis* MHOM/BR/pH8 strain were preserved in Schneider medium (Sigma-Aldrich, St Louis, MO, USA) supplemented with 20% fetal bovine serum (FBS) and 1% streptomycin/penicillin, at 26 °C, in a B.O.D incubator—J. Prolab, São José dos Pinhais, Brasil, model JP. 100 (LBCM/CPAM). The promastigote forms were used in the exponential growth phase in all stages of the experiment [80].

#### 4.2.2. Macrophage Cultive

The macrophages J774A.1 strain (ATCC TIB-67) was kept under cryopreservation. The thawing process started with the transfer of the cryopreserved content to a Falcon tube containing 5 mL of DMEM culture medium (Gibco^®^, Billings, MT, USA), supplemented with 10% inactivated fetal bovine serum, in addition to 1% non-amino acids essential oils and 1% gentamicin sulfate. The tube was centrifuged, and the cells were resuspended and transferred to a culture bottle kept in an incubator at 37 °C with 5% CO_2_ [81].

#### 4.2.3. Determination of the Inhibitory and Cytotoxic Effects in *L. amazonensis* and JJ74 Macrophage Cultures

To assess inhibitory or cytotoxic concentrations of the synthesized compound capable to eliminate 50% of the cells in the cultures of *L. amazonensis* amastigotes (IC_50_) and cytotoxic concentrations in JJ74 macrophage cultures (CC_50_), MTT [3-(4,5-dimethylthiazol-2-yl)-2,5-diphenyltetrazolium bromide] (Sigma-Aldrich) method was used [80]. In a 96-well-plate, the compound was diluted in the following concentrations: 0, 0.5, 1, 2, 4, 8, 16, 32, 64, 128, 256 μg mL^−1^. In parallel, 2.0 × 10^5^ axenic amastigotes and 1.0 × 10^5^ JJ74 macrophages were distributed per well, according to the assay in question. The plates were incubated for 24 h and 2 h, respectively, at 37 °C with 5% CO_2_. Then, 10 μL of MTT was added per well and the cultures were re-incubated for a period of 4 h. Subsequently, after the incubation time, 50 μL of DMSO was added to each well for the solubilization of the formazan crystals. The absorbances were read on a SpectraMAX GeminiXS ^®^ plate spectrophotometer (Molecular Devices LLC, San Jose, CA, USA) at a wavelength of 570 nm [82].

On the other hand, the methodology for evaluating the antipromastigote activity in *L. amazonensis* strains was based on the Neubauer chamber method, as described by Serafim et al. [12]. Promastigotes in the logarithmic growth phase were collected, counted, and diluted in Schneider medium (Sigma-Aldrich, St. Louis, MO, USA) supplemented with 20% FBS in a concentration of 1 × 10^6^ cells mL^−1^. Subsequently, the cells were incubated with different concentrations of the compound (10; 5; 2.5, 1.25; and 0.625 μg/mL). The negative control consisted of cells incubated only in Schneider medium, while the positive control comprehended the use of the commercial drug Amphotericin B^®^ (Cristália, Itapira, SP, Brazil). Culture growth was observed after 72 h of incubation at 25 °C by counting viable cells using a Neubauer chamber, and calculated using the following Equation (1):

No. Leishmania/mL = No. of cells counted × No. of Neubauer chamber quadrants × the dilution used × 10^4^ (Neubauer chamber correction factor) (1)

The concentration that inhibits 50% of parasite growth (IC_50_) was the parameter used to estimate growth inhibition. Therefore, IC_50_ was determined after 72 h of cultivation by linear regression analysis with SPSS 8.0 software for Windows. Each test was performed in 2 independent experiments, with different cultures, and in technical triplicate.

#### 4.2.4. Cytotoxicity Evaluation in Macrophages by Annexin V-FITC/PI Double Staining Assay

The cytotoxicity assay in JJ74 macrophages was performed the molecule through the method of Annexin V-FITC/PI double staining assay by flow cytometer BD LSRFortessa^TM^ (BDBiosciences, San Jose, CA, USA). This assay is based on the ability of annexin V to bind to phosphatidylserine, which is externalized in apoptotic events, and propidium iodide to bind to the DNA of cells that have lost membrane integrity. Thus, this study allows the detection of viable cells (Annexin–/PI–), early apoptotic cells (Annexin+/PI–), late apoptotic cells (Annexin+/PI+) and necrotic cells (Annexin–/PI+) [83,84].

The analyses took place in 96-well plates, and 4 × 10^4^ macrophages were distributed per well, with the addition of the molecule in concentrations of 1, 16 and 32 μg mL^−1^. These were incubated in DMEM (Gibco^®^, Billings, MT, USA) medium for two hours at 37 °C with 5% CO_2_. Subsequently, 2 μL of annexin + 2 μL PI was added to each well, followed by ten minutes of incubation. Lastly, the plates were centrifuged, with the removal of 100 μL of the supernatant and the addition of 200 μL of saline. Fluorescence-activated cells were analyzed in a flow cytometer. The analyses were performed in sextuplicate [85].

#### 4.2.5. Microbicidal Assay

The J774 macrophage cells were quantified and distributed (1.5 × 10^5^ cells/excavation) in 24 excavation-plates in DMEM medium, adding coverslips previously washed in each well to promote cell adhesion. The cells were incubated for 2 h at 37 °C with 5% CO_2_. Subsequent to the cell adhesion process, the excavations were washed with STF pH 7.2; 37 °C, followed by the addition of the amastigote forms of *L. amazonensis* (1.5 × 10^6^/excavation). To promote macrophage infection, these were incubated for 12 h at 37 °C with 5% CO_2_, in DMEM medium added with 10% Bovine Fetal Serum (Sigma Aldrich, St. Louis, MO, USA).

To remove the non-phagocyted Leishmanias, the excavations were washed three times with sterile FTS at 37 °C. The molecule was then incubated at the before-mentioned concentrations during a 2 h treatment period. Finally, the surplus medium was removed, the excavations were exposed to drying and fixed in methanol, being later stained with Giemsa 10% solution and evaluated by optical microscopy (1000×).

The microbicidal effect was evaluated by determining infection in 200 macrophages, being expressed as a product of the mean of phagocyted Leishmanias by the percentage of infected macrophages. To determine the estimated percentage of macrophages adhered to the coverslips, the cells present in 10% of the coverslips were quantified with the assistance of optical microscopy (400×) [86].

#### 4.2.6. Assessment of Cytokine Production by Macrophages

The quantification of cytokines IL-2, IL-4, IL-6, IL-10, IL-17A, INF-γ, and TNF-α was performed in the supernatant of cell cultures of J774 macrophages infected with *L. amazonensis* amastigotes, by cytometry bead array (CBA), according to the manufacturer’s instructions (BD Bioscience, Franklin Lakes, NJ, USA). Macrophages were adhered to a plate infected with *L. amazonensis* and treated with the compound under analysis at concentrations of 2, 4, and 8 μg mL^−1^. Uninfected and untreated macrophages were used as the negative control, while the untreated macrophages infected with *L. amazonensis* were used as the positive control. After the 24-h treatment period, 50 μL of the supernatant from each well was transferred to a conical tube, followed by the addition of 50 μL of the capture bead mixture and 50 μL of the detection reagent. A calibration curve containing the following concentrations of each cytokine was added to the experiment: 0 pg mL^−1^, 20 pg mL^−1^, 40 pg mL^−1^, 80 pg mL^−1^, 156 pg mL^−1^, 312.5 pg mL^−1^, 625 pg mL^−1^, 1250 pg mL^−1^, 2500 pg mL^−1^ and 5000 pg mL^−1^. The fluorescence intensity in each sample was captured on the BD LRS II FORTESSA flow cytometer using the DIVA program version 7 (BD Bioscience), and the fluorescence data were processed using the FCAP array program version 3 (BD Bioscience). The experiments were performed in triplicate [86].

#### 4.2.7. Evaluation of the Production of Reactive Oxygen and Nitrogen Species

The quantification of ROS and RNS was performed using the probe 2′,7′-dichlorodihydrofluorescein diacetate (DCF-DA) and 4-amino-5-methylamino-2′,7′-difluorofluorescein diacetate (DAF-FM diacetate), which produce, in the presence of reactive species, 2-7-dichlorofluorescein (DCF) and 4-amino-5-methylamino-2′,7′-difluorofluorescein (DAF-FM), respectively, which fluoresce and remain inside the cell. Macrophages were adhered to a plate infected with *L. amazonensis* and treated with the compound under analysis at concentrations of 2, 4, and 8 μg mL^−1^. Uninfected and untreated macrophages were used as the negative control, while the untreated macrophages infected with *L. amazonensis* were used as the positive control. After the 24-h treatment period, the plates were incubated for 30 min with the DCF-DA probe diluted in saline pH 7.2 and 1 h with the DAF-FM diacetate probe diluted in saline pH 7.2, respectively, in a humid chamber at 37 °C with 5% CO_2_, protected from light. Then, the cells were washed with saline, pH 7.2, at room temperature and resuspended with 200 µL of saline under the same conditions. The fluorescence intensity in each sample was captured on the BD LRS II FORTESSA flow cytometer using the DIVA program version 7 (BD Bioscience) and the mean fluorescence intensity (MFI) was obtained after processing the data in the FlowJoTM version 10.6.1. The experiment was performed in triplicate and the results were calculated as the difference in MFI in each group [86].

### 4.3. ctDNA Interaction Evaluation by UV–vis Absorption

The interaction in vitro of ACW-02 and calf thymus DNA (ctDNA) (Sigma Aldrich, Saint Louis, MO, USA) was conducted in 10 mM Tris-HCl buffer (pH 7.6), in a rectangular quartz cuvette with 1 cm path length at 25 °C. CtDNA concentration in Tris-HCl was defined as micromolar equivalents of the base pairs [87]. The derivative stock solution was prepared in DMSO (1 mM), and subsequently, diluted in Tris-HCl buffer, on concentrations ranging from 10 to 100 µM. Afterward, the absorption spectral titration experiment was performed by keeping the compound in a constant concentration (50 µM) and modifying DNA concentration (0–100 µM bp). The intrinsic binding constant (Kb) of the compound with ctDNA was calculated by fitting the data to Equation (2), in which coefficients of apparent (ε_a_), bound (ε_b_) and free extinction (ε_f_) were used [22]:

[DNA]/(ε_a_ − ε_f_) = (DNA)/(ε_b_ − ε_f_) *+* 1/Kb(ε_b_ − ε_f_) (2)

The data were fitted by using the software SigmaPlot 10.0. Plot fitting of [DNA]/(ε_a_ − ε_f_) vs. [DNA] utilized the Kb acquired from the percentage of the slope to the Y intercept [88].

### 4.4. Statistical Analysis

The normality of the variables was analyzed employing the Kolmogorov-Smirnov test and the homogeneity of the variances, using the Bartlett test. Paired *t*-test or Wilcoxon test were used to compare two normal or non-normal samples, respectively. For multiple comparisons, the ANOVA test was employed, for parametric or non-parametric data, respectively. Analyzes and graphical representations were performed using the Prism^®^ Software Package program (GraphPad ^®^, Boston, MA, USA, 1997). Differences were considered significant at *p* value < 0.05 [89].

### 4.5. Homology Modeling

Because there is no crystallographic structure of the cysteine protease B from *Leishmania amazonensis* (CPBla), it was decided to carry out a homology modeling and thus build the model similar to other works [90,91]. Initially, the amino acid sequence for the required cysteine protease was obtained from the National Center for Biotechnology Information Search database (NCBI, (https://www.ncbi.nlm.nih.gov/, accessed on 10 September 2022) [92], under the code AAP21894, consisting of 353 amino acids. Then, a search for proteins with structural identity was performed using the Basic local alignment search tool server (BLAST, https://blast.ncbi.nlm.nih.gov/Blast.cgi, accessed on 10 September 2022) [93]. Thus, the search revealed 100 homologous sequences, so the one with the best alignment was with the cruzain from *T. cruzi* (PDB: 1aim) [94], used as a template. Finally, the model was built using the Swiss-Model web software (https://swissmodel.expasy.org/, accessed on 10 September 2022) [95] and validated through the Ramachandran graph generated by the SAVES web software (https://saves.mbi.ucla.edu/, accessed on 12 September 2022) [96]. Finally, the developed model was aligned with the original PDB (1aim) using the PyMol software, and the Root Mean Square deviation (RMSD) value was calculated for the final validation of the model [97].

### 4.6. Molecular Docking Studies

The studied structures were first treated based on the semi-empirical theory at the PM6 level using the Spartan 14 software. After being optimized, they were submitted to the study of molecular docking through the Gold 5.8.1 program. The structure of the dodecamer (PDB ID: 1Z3F) was used as a DNA model, trypanothione reductase from *L. infantum* (PDB ID: 4APN), topoisomerase I from *L. donovani* (PDB ID: 2B9S), and 14-alpha-ester demethylase from *L. infantum* (PDB ID: 3L4D) were selected and obtained from the Protein Data Bank, in view of the homology between *L. amazonensis*, *L. donovani* and *L. infantum* species genetic sequence [10,98,99,100,101,102]. To validate the study, redocking for targets were performed with co-crystallized ligands. Through the docking results provided by GOLD software, the intermolecular interactions were determined and used as a starting point to measure the affinity of the ligand for the interaction site. The conformation with the best score was analyzed using the Pymol 2.3.2 software, evaluating the distance and connection with the receptor for the outcomes of molecular modeling studies [103].

### 4.7. Molecular Dynamics Simulations 

The complexes of ACW-02 with TryR, Topo I, CYP51, and CPBLa obtained in molecular docking were used in molecular dynamic (MD) simulations using the GROMACS*^®^* software. Initially, charge and hydrogens were added, and water molecules were removed from the complexes using the DockPrep available in Chimera^®^ software. Next, the CHARMM36 force field and TIP3P solvation method were selected. At the same time, Ligand topologies were generated using the web software SwissParam (http://www.swissparam.ch/, accessed on 29 September 2022) [104]. Then, a 1.0 nm triclinic box was created, and ions and water were added in a physiological concentration (0.15 M). The system equilibrations were started, with 10,000 steps by the conjugate gradient method, and subsequently, a total minimization at 20,000 steps. With the minimization of system energy, the NvT (constant Number of particles, Volume, and Temperature) and NpT (constant Number of particles, Pressure, and Temperature) balances were employed in 300 K at 10 ns. Finally, the simulation was performed at 100 ns with the system equilibrated. After obtaining the trajectory files, the Root-mean-square deviation (RMSD), root-mean-square fluctuation (RMSF), radius of gyration (R_g_), and H-bond plots were generated using the Xmgrace*^®^* software. This protocol is according to other works performed by our research team [105,106,107].

### 4.8. MM-PBSA Calculations

After the molecular dynamic simulations, the trajectory files were used to calculate the free binding energy of the ligand in complex with the macromolecule through the Molecular Mechanics/Poisson–Boltzmann Surface Area (MM-PBSA) method. This method is used frequently in high-throughput virtual screenings to reduce the incidence of false positives [108]. In this way, it’s possible to calculate the Gibbs free-biding energy (Δ*G*_binding_) during the simulation, using van der Waals and electrostatic (unbound) interactions [109]. The difference between the free energy of complex protein-ligand (*G*_cpx_) and unbound protein and ligand (*G*_rec_) are used to calculate the Δ*G*_binding_. Finally, this value is calculated based on the sum of the changes in the solvation entropy (−*T*Δ*S*_sol_), binding energy (Δ*E*_bind_), and conformational entropy (−*T*Δ*S*_conf_) (Equation (3)) [110]. Thus, Δ*G*_binding_ and interactions parameters calculated by MM-PBSA were performed using the *g_mmpbsa* tool [108] accoupled in the GROMACS*^®^* software with the trajectory files of the molecular dynamics simulation. The Δ*G*_binding_ values were obtained as the average free-interaction and solvation energies [109].

Δ*G*_binding_ = *E*_binding_ − *T*Δ*S*_sol_ − *T*Δ*S*_conf_ (3)

## 5. Conclusions

In conclusion, a novel active antileishmanial compound has been synthesized, with high selectivity for amastigote forms of *L. amazonensis* and non-toxicity for macrophages up to the highest concentration tested. Additionally, the compound appeared to perform immunomodulatory activity toward macrophages’ infection management due to the down expression of the pro-Leishmania cytokines IL-10 and IL-6. Furthermore, evaluated in silico and in vitro, the compound ACW-02 presented the ability to bind with DNA, suggesting this as one of the potential targets responsible for the activity performed. Additional theoretical studies of molecular docking, dynamics and MM-PBSA calculations in *Leishmania* targets contributed to a possible mechanistic elucidation and justification of the selectivity of the compound for the parasite cell by topoisomerase IB and CPBLa inhibition. Therefore, this preliminary study supports the potential antiparasitic action of this acridine compound, encouraging further studies involving this proposal.

## Figures and Tables

**Table 1:** Data on 50% growth inhibition of *L. amazonensis*, cytotoxic concentration on macrophages (CC_50_), concentration that induces stimulatory effect on macrophages (EC_50_), and selectivity index (SI), of ACW-02 and positive control Amphotericin B.

COMPOUND	IC_50AMA_ (µg mL^−1^)	IC_50PRO_ (µg mL^−1^)	CC_50_ (µg mL^−1^)	EC_50_ (µg mL^−1^)	SI (CC_50_/IC_50AMA_)
**ACW-02**	6.57 ± 0.04	94.97 ± 0.06	>256.00	9.46 ± 0.06	>38.94
**AMPHOTERICIN B**	2.94 ± 0.07	1.41 ± 0.003	3.09 ± 0.01	2.80 ± 0.0093	1.05

**Table 2:** Data on ACW-02 microbicidal effect assay.

ACW-2 (µg mL^−1^)	Infected Cells (%)	Mean of *Leishmanias*	Infection Rate (%)
**0**	22.00	2.40	53.00
**1**	12.00	1.20	14.50
**16**	11.50	1.20	13.50
**32**	11.50	1.80	21.00

**Table 3:** UV–vis absorption data of triazolidine acridine in the absence and presence of ctDNA.

Compound	λ_max_ Free (nm)	λ_max_ Bound (nm)	∆λ (nm)	Hyperchr ^a^ (%)	Hypochr ^b^ (%)	Kb (M^−1^)
**ACW-02 + DNA**	460	461	1	47.53	-	1.17 × 10^6^

^a^ Hyperchromicity for complexes formed by compounds and 100 μM of ctDNA in comparison to free ligands. ^b^ Hypochromicity for complexes formed by compounds and 100 μM of ctDNA in comparison to free ligands. Kb: affinity constant.

**Table 5:** Binding energy and interaction parameters calculated by MM-PBSA.

	2B9S (Topo I)	CPBLa
**ΔG_bing_ (KJ/mol)**	−42.17 ± 21.36	−103.13 ± 8.11
**SASA Energy (KJ/mol)**	−10.66 ± 1.69	−13.15 ± 0.78
**Polar solvation energy (KJ/mol)**	65.34 ± 23.63	61.09 ± 5.20
**Electrostatic energy (KJ/mol)**	−1.87 ± 19.59	0.68 ± 3.60
**Van der Waals energy (KJ/mol)**	−94.98 ± 25.52	−151.75 ± 7.79

## Data Availability

Data is contained within the article and Supplementary Materials.

## Supplementary Materials

The following supporting information can be downloaded at: https://www.mdpi.com/article/10.3390/ph16020204/s1, Figure S1: ^1^H NMR spectrum of ACW-02 (500 MHz, DMSO-d6); Figure S2: ^13^C NMR spectrum of ACW-02 (125 MHz, DMSO-d6); Figure S3: Extended Mass spectrum of ACW-02 by MALDI-TOF; Figure S4: Infrared (IR) of ACW-02; Figure S5: Annexin V-FITC/PI assay with control group; Figure S6: Effect of compound ACW-02 on macrophages. B7–B12—1 µG mL^−1^. C1–C6—16 µG mL^−1^. C7–C12—32 µG mL^−1^; Table S1: Descriptive statistics of ACW-02 in vitro experiments; Figure S7: Molecular docking of ACW-02 (A) on trypanothione reductase from *L. infantum* (PDB ID: 4APN); Figure S8: Molecular docking of ACW-02 (A) on sterol 14-alpha demethylase (CYP51) from *L. infantum* (PDB ID: 3L4D); Figure S9: Molecular docking of ACW-02 on topoisomerase 1 (TOP I) from *L. donovani* (PDB ID: 2B9S); Figure S10: Molecular docking of ACW-02 on cysteine protease B from *L. amazonensis* (CPBLa).
