# Supplementary material for: ACW-02 an Acridine Triazolidine Derivative Presents Antileishmanial Activity Mediated by DNA Interaction and Immunomodulation
Source: Pharmaceuticals (Basel). 2023 Jan 29;16(2):204. doi: 10.3390/ph16020204 (PMC9967605; doi:10.3390/ph16020204)
Supplement: Supplementary file 1 [file pharmaceuticals-16-00204-s001.zip › pharmaceuticals-2156523-supplementary.pdf]

Experimental Section 1 (Structural Characterization)

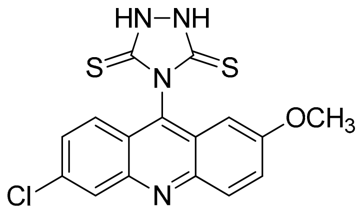

4-(6-chloro-2-methoxyacridin-9-yl)-1,2,4-triazolidine-3,5-dithione (ACW-02)

Orange crystals. Formula: $C_{16}H_{11}ClN_4OS_2$; M.W.: 374.8610 g mol^{-1}; Yield: 50%; Melting point: 282-284 ºC; R*f*: 0.50 (*n*-hexane/EtOAc 7:3). ^{1}H NMR (500 MHz, DMSO-*d*6): δ 3.88 (3H, *s*, OCH3); 7.36 (1H, *dd*, J = 2.05 Hz; J = 9.05 Hz, H-12); 7.52 (1H, *dd*, J = 2.9 Hz; J = 9.05 Hz, H-01); 7.62 (1H, *d*, J = 9.1 Hz, H-06); 7.65 (1H, *d*, J = 2 Hz, H-14); 8.25 (1H, *d*, J = 2.9 Hz, H-03); 8.83 (1H, *d*, J = 2.9 Hz, H-11); 12.79 (2H, *s*, NH). ^{13}C NMR (100 MHz, DMSO-*d*6): δ 194.6459 (C-19, C-16); 156.3423 (C-13); 138.5071 (C-4); 136.2716 (C-8); 132.4865 (C-3); 131.3356 (C-2); 130.4602 (C-5); 127.5769 (C-9); 126.3238 (C-6); 126.9683 (C-12); 120.9024 (C-1); 117.8267 (C-11); 108.4340 (C-14); 55.8649 (OCH_3). IR (KBr, cm^{-1}): 3242-3147 (N-H); 3085-3056 (C-H_{Ar}); 1621-1482 (C=C_{Ar}); 1482 (N-C=S); 1208 (C=S); 1197 (C=C-O). MALDI-TOF MS m/z $[M+H]^+$: calculated = 374.0063; found = 375.0096.

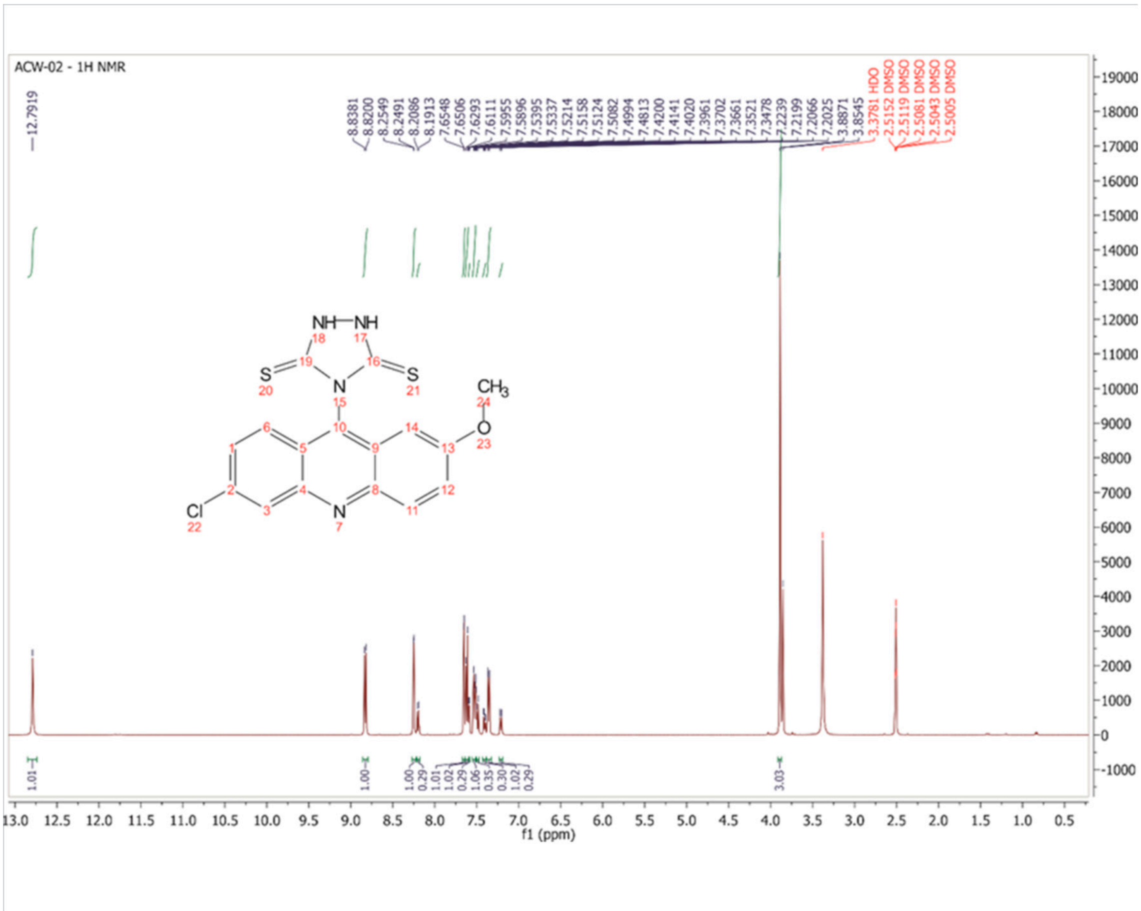

Figure S1. ^{1}H NMR spectrum of ACW-02 (500 MHz, DMSO-*d6*)

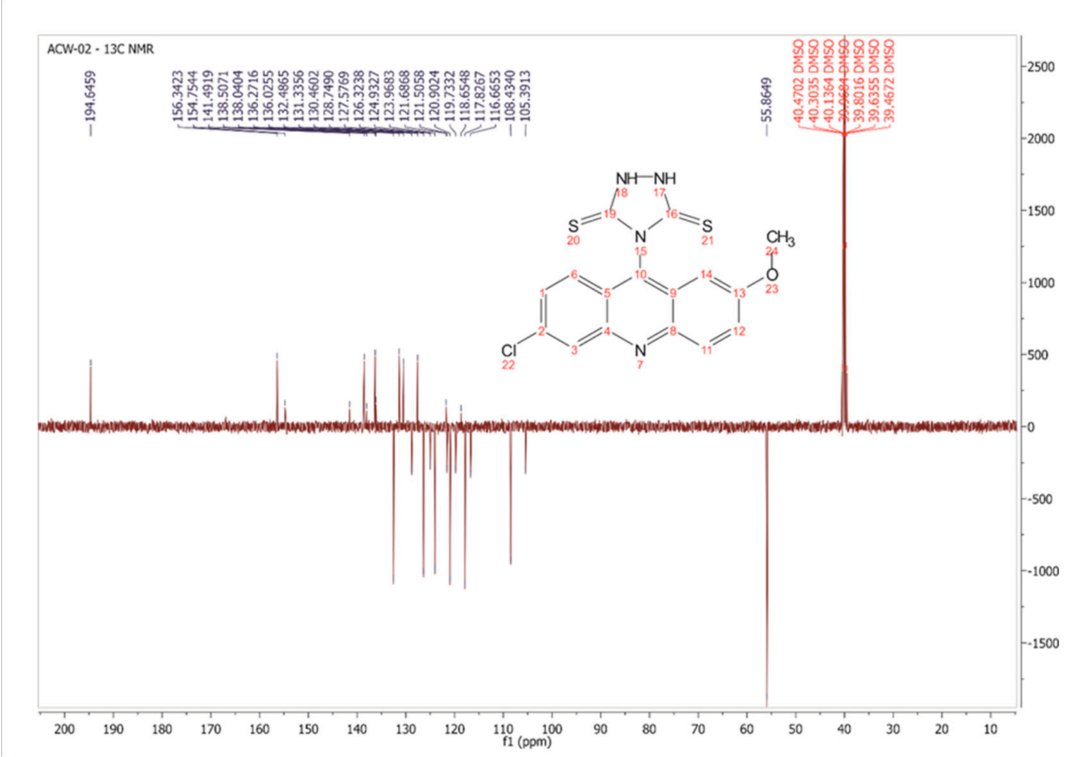

Figure S2. ^{13}C NMR spectrum of ACW-02 (125 MHz, DMSO-*d6*)

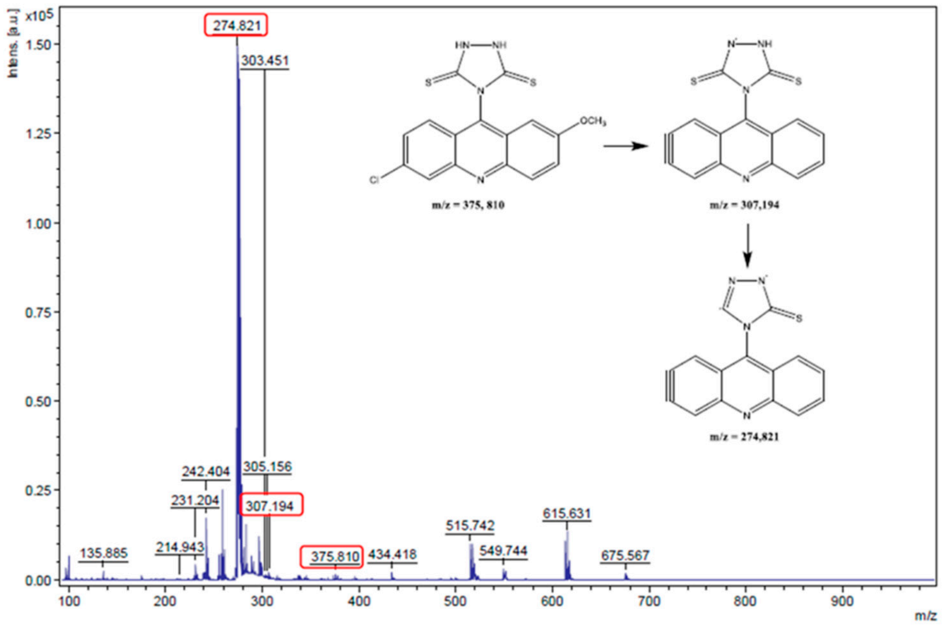

Figure S3. Extended Mass spectrum of ACW-02 by MALDI-TOF

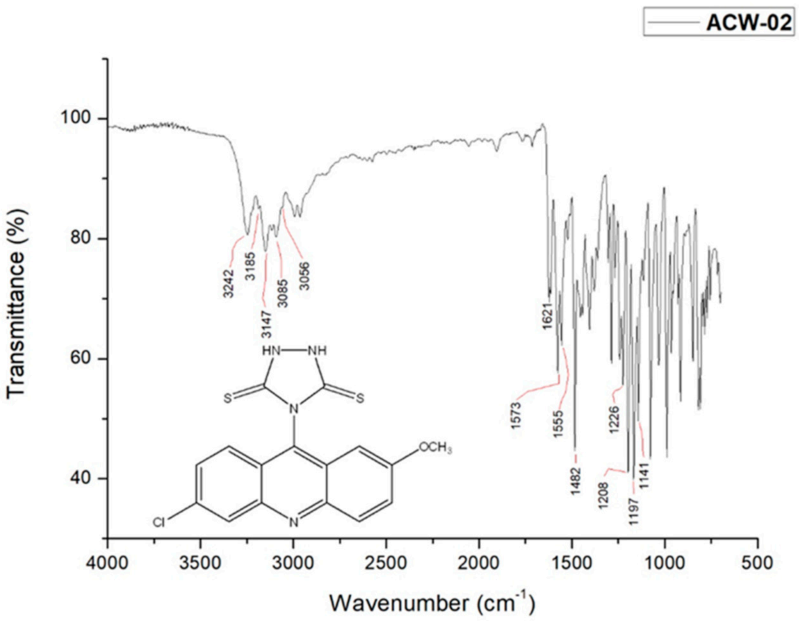

Figure S4. Infrared (IR) of ACW-02

Experimental Section 2 (*In vitro* studies)

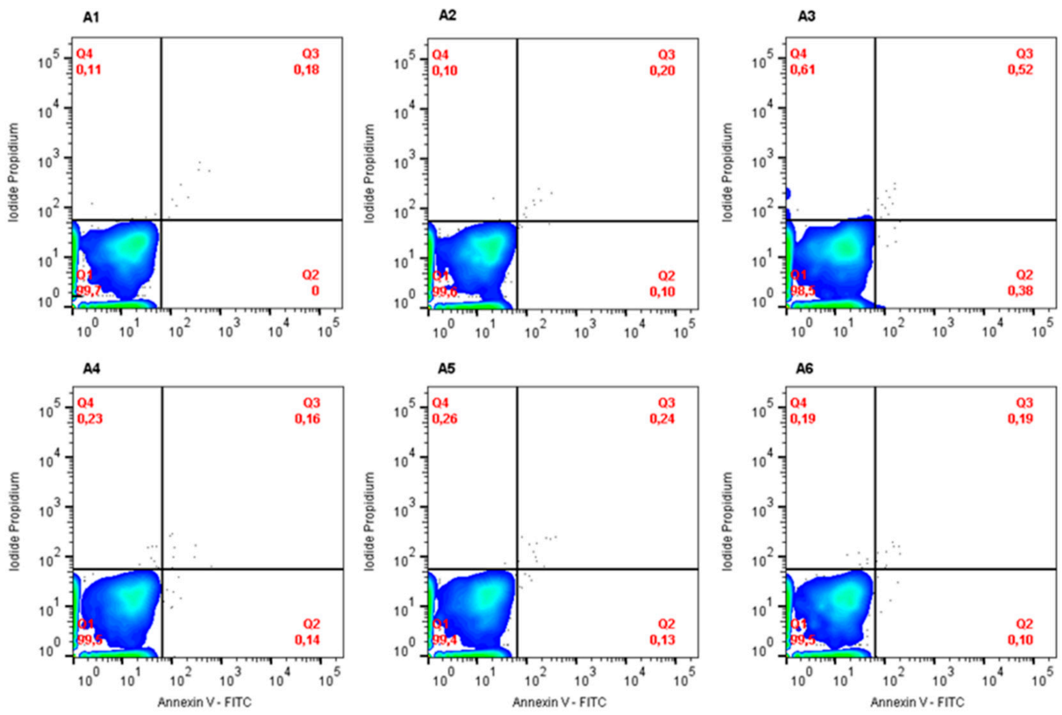

Figure S5. Annexin V-FITC/PI assay with control group

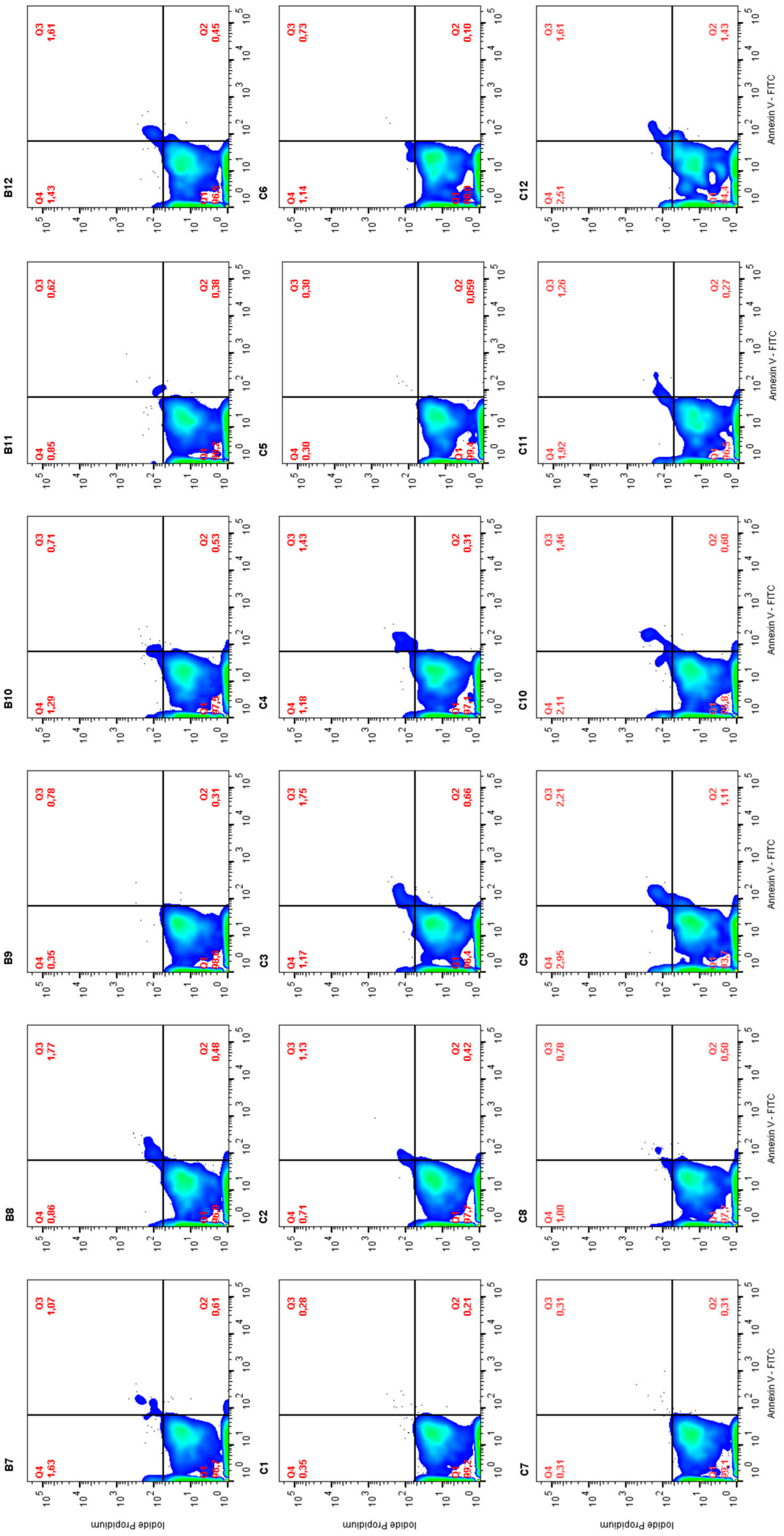

Figure S6. Effect of compound ACW-02 on macrophages. B7-B12 – 1 µG mL^{-1}. C1-C6 – 16 µG mL^{-1}. C7-C12 – 32 µG mL^{-1}.

Table S1. Descriptive statistics of ACW-02 *in vitro* experiments.

Experiment	Concentration/condition evaluated (μg mL^{-1})	Mean	Standard error of mean (±)
Cytotoxicity in macrophages (3.3)	0 – Early Apoptosis	0,05667	0,02603
Cytotoxicity in macrophages (3.3)	0 – Later Apoptosis	0,1667	0,08950
Cytotoxicity in macrophages (3.3)	0 – Necrosis	0,3067	0,008819
Cytotoxicity in macrophages (3.3)	1 – Early Apoptosis	0,4600	0,04351
Cytotoxicity in macrophages (3.3)	1 – Later Apoptosis	1,093	0,1995
Cytotoxicity in macrophages (3.3)	1 – Necrosis	1,068	0,1917
Cytotoxicity in macrophages (3.3)	16 – Early Apoptosis	0,2933	0,09120
Cytotoxicity in macrophages (3.3)	16 – Later Apoptosis	0,9367	0,2464
Cytotoxicity in macrophages (3.3)	16 – Necrosis	0,8083	0,1690
Cytotoxicity in macrophages (3.3)	32 – Early Apoptosis	0,7033	0,1904
Cytotoxicity in macrophages (3.3)	32 – Later Apoptosis	1,272	0,2706
Cytotoxicity in macrophages (3.3)	32 – Necrosis	1,800	0,3999
Cytokine production (3.5)	Basal - TNF	126,2	0,3926
Cytokine production (3.5)	Leish- TNF	91,51	2,934
Cytokine production (3.5)	2- TNF	23,07	1,457
Cytokine production (3.5)	4- TNF	20,91	0,2558
Cytokine production (3.5)	8- TNF	21,90	0,9601
Cytokine production (3.5)	Basal – INF	6,170	0,01155
Cytokine production (3.5)	Leish – INF	5,723	0,07623
Cytokine production (3.5)	2 – INF	5,113	0,09838
Cytokine production (3.5)	4– INF	4,947	0,2050
Cytokine production (3.5)	8– INF	5,313	0,2410
Cytokine production (3.5)	Basal – IL-2	7,583	0,01453
Cytokine production (3.5)	Leish – IL-2	7,193	0,02333
Cytokine production (3.5)	2 – IL-2	7,300	0,06658
Cytokine production (3.5)	4 – IL-2	7,080	0,3011
Cytokine production (3.5)	8 – IL-2	7,220	0,07572
Cytokine production (3.5)	Basal – IL-10	2,533	0,2794
Cytokine production (3.5)	Leish– IL-10	5,163	0,2245
Cytokine production (3.5)	2– IL-10	1,797	0,9308

Cytokine production (3.5)	4– IL-10	1,500	0,4864
Cytokine production (3.5)	8– IL-10	1,213	0,6271
Cytokine production (3.5)	Basal – IL-4	7,220	0,4013
Cytokine production (3.5)	Leish– IL-4	7,347	0,1367
Cytokine production (3.5)	2– IL-4	7,140	0,1480
Cytokine production (3.5)	4– IL-4	6,457	0,1729
Cytokine production (3.5)	8– IL-4	6,773	0,1224
Cytokine production (3.5)	Basal– IL-6	7,770	0,06928
Cytokine production (3.5)	Leish– IL-6	7,490	0,02309
Cytokine production (3.5)	2– IL-6	5,753	0,2348
Cytokine production (3.5)	4– IL-6	6,230	0,1604
Cytokine production (3.5)	8– IL-6	5,840	0,1539
Cytokine production (3.5)	Basal– IL-17A	5,950	0,02887
Cytokine production (3.5)	Leish– IL-17A	6,413	0,02333
Cytokine production (3.5)	2– IL-17A	5,867	0,1970
Cytokine production (3.5)	4– IL-17A	5,720	0,1856
Cytokine production (3.5)	8– IL-17A	5,780	0,2892
Reactive oxygen and nitrogen species (3.6)	Basal – ROS	3852	318,6
Reactive oxygen and nitrogen species (3.6)	Leish– ROS	7933	442,5
Reactive oxygen and nitrogen species (3.6)	2– ROS	8006	77,83
Reactive oxygen and nitrogen species (3.6)	4– ROS	7293	225,0
Reactive oxygen and nitrogen species (3.6)	8– ROS	7658	133,7
Reactive oxygen and nitrogen species (3.6)	Basal– RNS	1193	26,27
Reactive oxygen and nitrogen species (3.6)	Leish– RNS	1055	64,67
Reactive oxygen and nitrogen species (3.6)	2– RNS	1087	4,041
Reactive oxygen and nitrogen species (3.6)	4– RNS	1047	46,61
Reactive oxygen and nitrogen species (3.6)	8– RNS	1016	14,18

Experimental Section 3 (*In silico* studies)

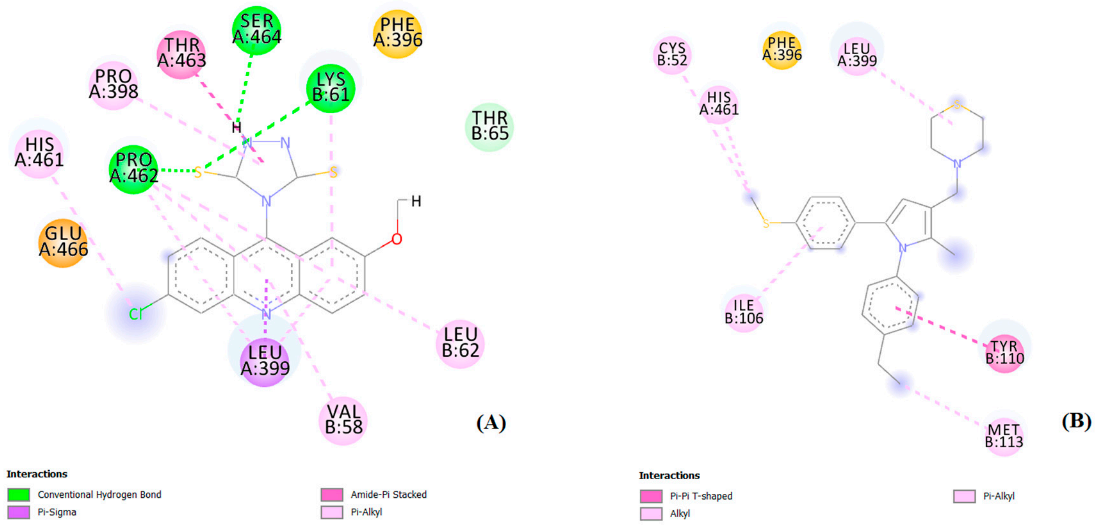

Figure S7. Molecular docking of ACW-02 (A) on trypanothione reductase from *L. infantum* (PDB ID: 4APN). A direct docking comparison was performed with a diarylpyrrole derivative (B), complexed in the crystallographic model used; this one has competitive mode inhibition reported to the TryR site. Types of interactions with the biological receptor are highlighted above for the respective compounds studied.

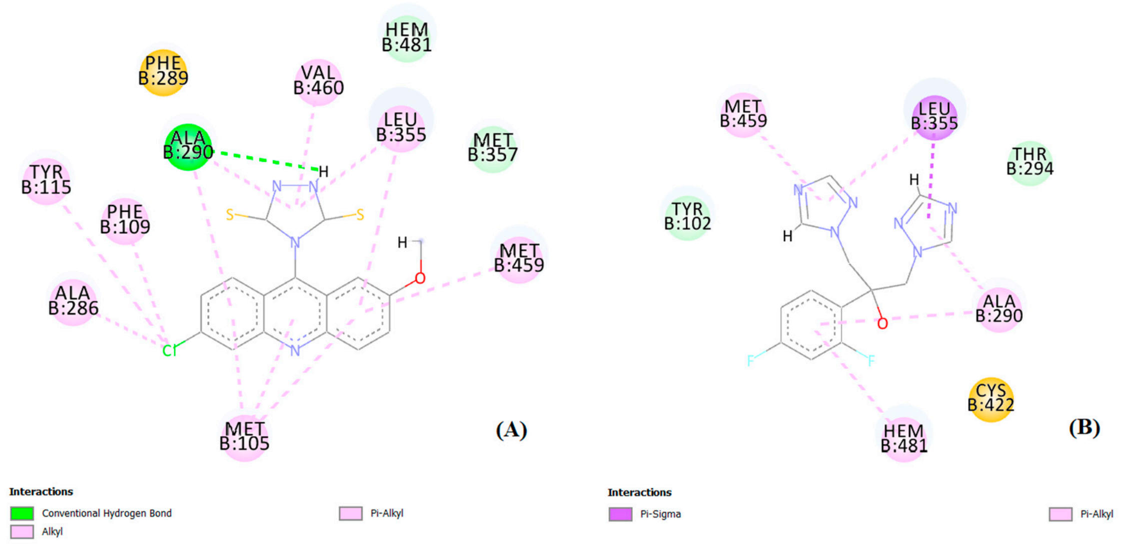

Figure S8. Molecular docking of ACW-02 (A) on sterol 14-alpha demethylase (CYP51) from *L. infantum* (PDB ID: 3L4D). A direct comparison of docking was performed with a known catalytic inhibitor of CYP51, fluconazole (B). Types of interactions with the biological receptor are highlighted above for the respective compounds studied.

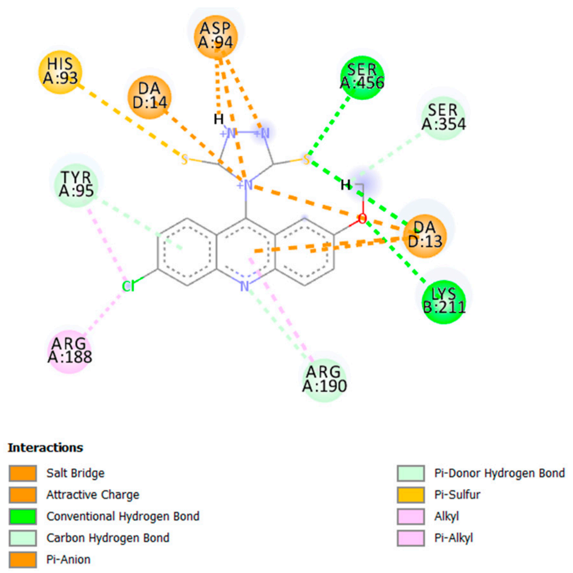

Figure S9. Molecular docking of ACW-02 on topoisomerase 1 (TOP I) from *L. donovani* (PDB ID: 2B9S).

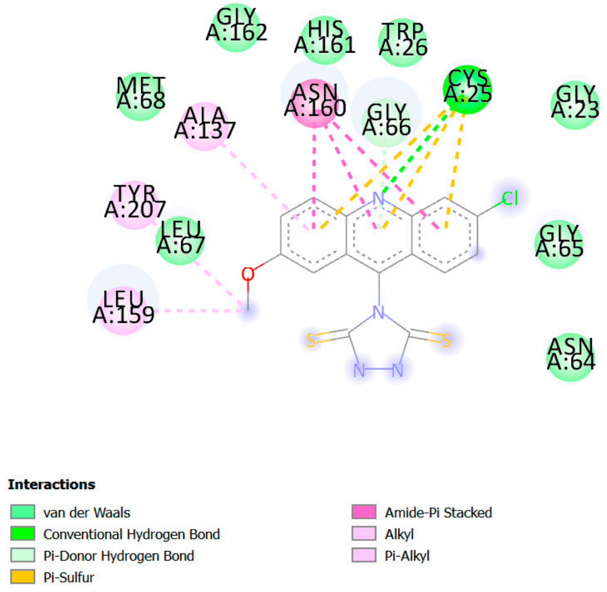

Figure S10. Molecular docking of ACW-02 on cysteine protease B from *L. amazonensis* (CPBLa).
